# Odontogenic cutaneous sinus tract associated with a mandibular second molar having a rare distolingual root: a case report

**DOI:** 10.1186/s13005-015-0072-y

**Published:** 2015-04-17

**Authors:** Jun Tian, Guobin Liang, Wenting Qi, Hongwei Jiang

**Affiliations:** Department of Operative Dentistry and Endodontics, Guanghua School of Stomatology, Affiliated Stomatological Hospital, Guangdong Province Key Laboratory of Stomatology, Sun Yat-sen University, Guangzhou, China; Department of Prosthodontics, Guanghua School of Stomatology, Affiliated Stomatological Hospital, Guangdong Province Key Laboratory of Stomatology, Sun Yat-sen University, Guangzhou, China

**Keywords:** Cone-beam computed tomography, Distolingual root, Mandibular second molar, Odontogenic cutaneous sinus tract, Sodium hypochlorite accident

## Abstract

**Introduction:**

Odontogenic cutaneous sinus tracts are often misdiagnosed as lesions of non-odontogenic origin, leading to the treatment of patients with unnecessary and ineffective therapies. Sinus tracts of endodontic origin usually respond well to endodontic therapy. However, root canal treatment of mandibular molars with aberrant canal anatomy can be diagnostically and technically challenging. Herein we present a patient with a cutaneous odontogenic sinus tract in the right submandibular area.

**Case report:**

A 23-year-old Chinese female patient presented with a cutaneous odontogenic sinus tract that was initially misdiagnosed as a sebaceous cyst. The patient had undergone surgical excision and traditional Chinese medical therapy before endodontic consultation. With the aid of cone beam computed tomography (CBCT), it was confirmed that the causative factor of the cutaneous odontogenic sinus tract was chronic periapical periodontitis of the right mandibular second molar, which had a rare and curved distolingual root. The resolution of the sinus tract and apical healing was accomplished following nonsurgical root canal treatment.

**Conclusion:**

A dental aetiology must be included in the differential diagnosis of cutaneous sinus tracts in the neck and face. Elimination of odontogenic cutaneous sinus tract infection by endodontic therapy results in resolution of the sinus tract without surgical excision or systemic antibiotic therapy. This case report also indicates that CBCT imaging is useful for identifying the tooth involved, ascertaining the extent of surrounding bone destruction and accurately managing the aberrant canal morphology.

## Introduction

Odontogenic cutaneous sinus tracts are rare dermatoses that occur because of chronic dental draining infections, especially apical periodontitis [1,2]. The most common locations for extraoral sinus tracts are the mandibular angles, chin and cheeks [3,4]. Extraoral fistulas typically present as erythematous, symmetrical, crusting, smooth and non-tender nodules with periodic drainage [5]. However, the dermal lesions are non-specific and can also present as abscesses, gummas, cysts, scars and ulcers [2]. Common dental causes of odontogenic sinus tracts include endodontic or periodontal infections, trauma, retained roots and residual chronic infection of the jaws [6-8]. The sinus tracts are most frequently associated with mandibular teeth, which have been documented in 80 ~ 87% of the reported cases [2,9]. However, only 50% of the patients experienced dental pain and the involved teeth are not always tender to percussion [4]. Additionally, the draining sinus tracts may be located at a distance from the origin of infection [8]. Therefore, odontogenic cutaneous sinus tracts are often misdiagnosed as lesions of non-odontogenic origin by surgeons and dermatologists, leading to unnecessary antibiotic or surgical therapies and the chronic persistence of the lesion [10]. Elimination of dental infection through endodontic treatments or tooth extraction is vital for the management of cutaneous sinus tracts [5].

This case report presents an odontogenic cutaneous sinus tract that was initially misdiagnosed as a non-odontogenic lesion. With the aid of cone beam computed tomography (CBCT), we identified the cause to be the right mandibular second molar with a rare and severe curved distolingual root.

## Case report

A 23-year-old Chinese female patient was referred to the Department of Conservative Dentistry and Endodontics, Guanghua School of Stomatology, Affiliated Stomatological Hospital, Sun Yat-sen University, Guangzhou, P.R. China, in order to verify a possible dental cause for a skin lesion. The patient’s chief complaint was the presence of a slightly stiff nodule in the right submandibular region, which had been periodically discharging pus for one year. She reported that the lesion had been previously diagnosed as a sebaceous cyst and had been surgically removed by a surgeon. Following its recurrence, for about seven months, she had undergone multiple failed regimens of traditional Chinese medical therapies. Finally, she was referred to a dermatologist and a dental aetiology was suspected. There was no history of dental pain, sensitivity or restricted mouth opening during the previous year. The patient’s medical history revealed no significant insights.

**Figure 1:**
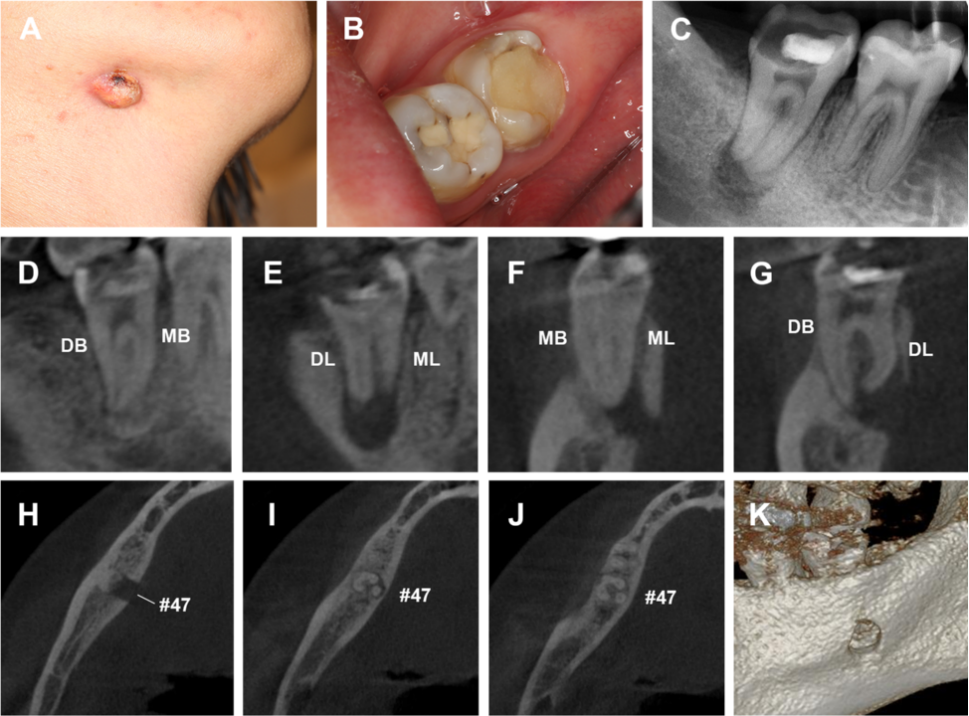
Preoperative clinical and radiographic examinations. **(A)** An extraoral cutaneous sinus tract presenting as an erythematous, smooth and non-tender nodule with crusting about 1 cm in diameter in the right submandibular region. **(B)** Preoperative clinical view of the right mandibular second molar with extensive glass-ionomer restoration. **(C)** A pretreatment radiograph of tooth #47 revealing obvious periapical radiolucency. CBCT imaging of the sagittal **(D** and **E)**, coronal **(F** and **G)** and axial view **(H-J)** showing the extent of bone loss around the apexes and the distolingual (DL) root with curvature in the coronal third and additional buccal curvature from the middle third to the apical third. **(K)** The 3D - reconstruction of the CBCT showing the perforation of the lingual cortical plate. DB, distobuccal; MB, meisobuccal; DL, distolingual; ML, meisolingual.

Extra-oral examination revealed an extra-orally draining sinus and an erythematous, smooth, non-tender and slightly stiff nodule with crusting about 1 cm in diameter in the right submandibular region (Figure 1A). No apparent facial swelling was observed. Intraoral examination revealed that the right mandibular second molar (#47) had been restored with glass ionomer cement (Figure 1B), was non-tender to percussion and had grade 1 mobility. Periodontal probing around the tooth revealed pocket depth within physiological limits with no intraoral sinus. Periapical radiography revealed the close proximity of the ionomer restoration to the pulp chamber and the presence of a periapical radiolucency associated with the root apexes of #47 (Figure 1C). The CBCT scan clearly demonstrated a well-defined periapical radiolucency, about 8.5 * 7.0 * 6.5 mm wide, perforating the lingual cortical plate. The tooth (#47) was found to contain three roots (mesial, distobuccal and distolingual root) and four root canals. Bone resorption involving the buccal cortex in the middle half of the roots was observed. The coronal view of the CBCT scan showed that the distolingual root was severely curved buccolingually (Figure 1D-K). As per the classification proposed by De Moor et al., [11] tooth #47 in our study belonged to type III with curvature in the coronal third and additional buccal curvature from the middle third to the apical third of the root. Based on the clinical and radiographic examinations, a diagnosis of chronic periapical periodontitis of the right mandibular second molar with cutaneous sinus tract was reached. Nonsurgical endodontic treatment of the tooth was scheduled.

**Figure 2:**
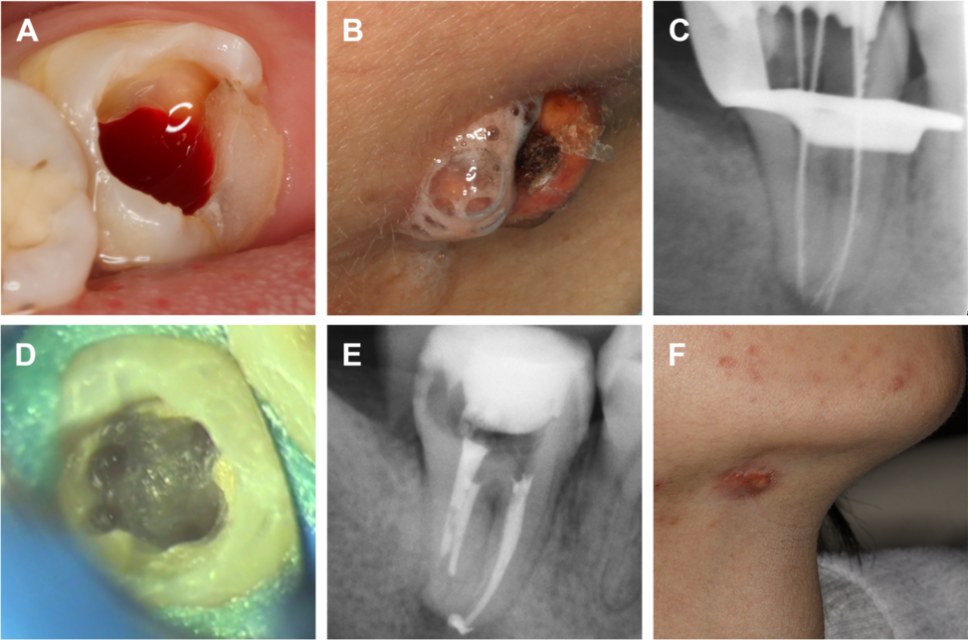
Clinical and radiographic records during the treatment. **(A)** Plentiful errhysis originating from the pulp chamber during and after accessing the cavity. **(B)** 3% hydrogen peroxide along with bloody pus leaking from the extra-oral drainage sinus during irrigation. **(C)** Working length determination. **(D)** Floor of the pulp chamber showing four orifices after preparation. **(E)** The root canals were packed with Vitapex after preparation. **(F)** The resolution of the sinus tract after dressing for one month.

Under local anesthesia, a trapezoidal endodontic access opening was established, resulting in copious amounts of dark red blood exuding from the pulp chamber (Figure 2A). The four root canals, namely the mesiobuccal (MB), meisolingual (ML), distobuccal (DB) and distolingual (DL) canal, were located under a dental operating microscope (DOM) following clearance of the errhysis. Patency was achieved using an ISO size #8 or #6 stainless steel K-file (Mani, Japan). Irrigation of canals with 3% perhydrol using a 27-gauge needle resulted in bloody pus discharge from the extra-oral sinus (Figure 2B). The canals were then dressed with calcium hydroxide paste.

Three days later, on the second visit, the tooth was restored with flowable composite resin (3M Dental Products, MN, USA) and isolated with a rubber dam under local anesthesia. Working lengths were determined with an electronic apex locator, Raypex5 (VDW, Munich, Germany), and confirmed by diagnostic radiography (Figure 2C). All canals were cleaned and shaped with Mtwo (VDW, Munich, Germany) and TF (Sybron Endo, Orange, CA) rotary NiTi instruments. The canals were irrigated using an endodontic syringe (Navy Tip; Ultradent, South Jordan, UT, USA) with 17% ethylenediamine tetra-acetic acid (EDTA) and 2.5% sodium hypochlorite (NaOCl) between each use of the files. The four canals were finally enlarged to Mtwo 20#06 and then to TF 25#08 (Figure 2D). The canals were packed with Vitapex (Neo Dental International Inc., WA, USA) (Figure 2E).

**Figure 3:**
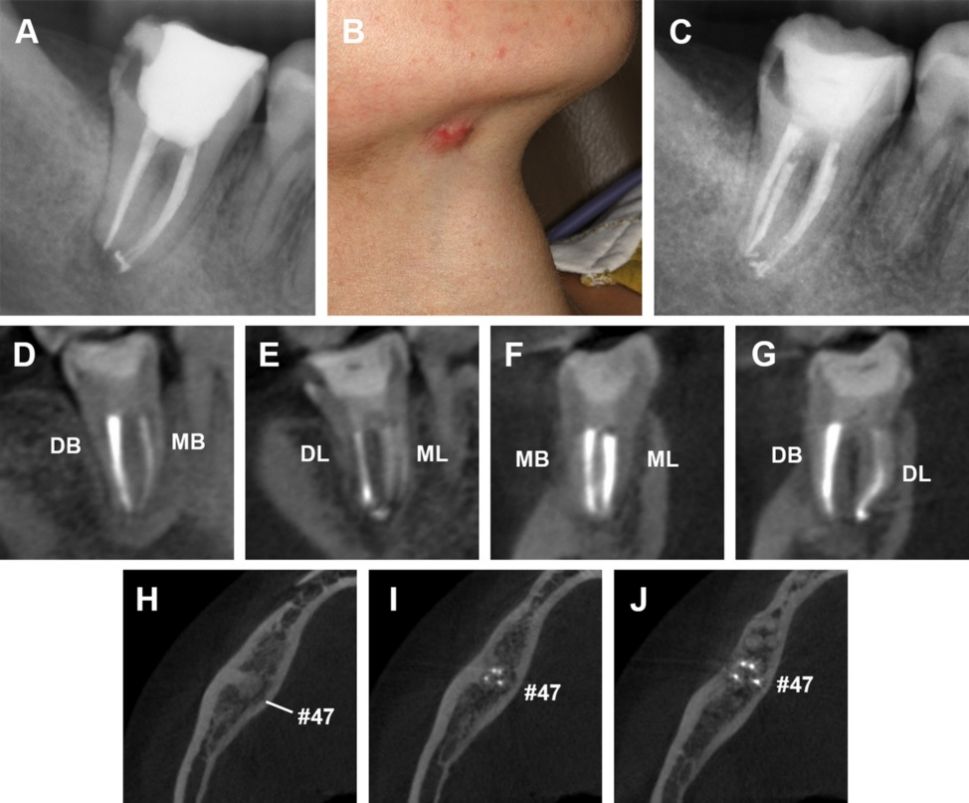
Postoperative radiographs of endodontic treatment and follow-up after five and nine months. **(A)** Post treatment periapical radiograph revealing that the obturation is of good quality. At the five-month follow-up, the extra-oral view **(B)** shows that the sinus tract had disappeared but left a scar in the right mandibular region. The periapical radiograph **(C)** shows the healing of periapical tissues. After nine months, postoperative CBCT images of the sagittal **(D** and **E)**, coronal **(F** and **G)** and axial view **(H**-**J)**view confirmed the apical healing and adequate obturation. DB, distobuccal; MB, meisobuccal; DL, distolingual; ML, meisolingual.

At the next visit, about one month later, the extra-oral sinus tract had healed and no purulent discharge was noted (Figure 2F). The canals were obturated using the continuous wave obturation technique with warm gutta-percha (Sybron Endo, Orange, CA) and AH Plus sealer (Dentsply, Maillefer, USA). The postoperative radiograph revealed a successful obturation (Figure 3A). At the five-month follow-up period, the sinus tract had disappeared but a scar from the surgery was left on the right submandibular area (Figure 3B). A radiograph confirmed adequate obturation and resolution of the periapical tissues (Figure 3C). Postoperative CBCT images after nine months confirmed the apical healing and adequate obturation (Figure 3D-J). The patient was subsequently advised to receive an appropriate coronal restoration.

## Discussion

Dental pathosis is the most common cause of cutaneous sinus tracts in the face and neck region and should be the primary suspect in differential diagnosis [4,12]. Other aetiology include osteomyelitis, pyogenic granuloma, salivary gland and duct fistulas, congenital sinus tract, infected cyst, deep mycotic infection and some skin lesions such as pustules, furuncles, foreign-body lesions, malignancy and granulomatous disorders [5,13].

Odontogenic cutaneous sinus tracts typically arise from periapical infections around the root apices as a result of pulpal necrosis, nearby caries or traumatic injury. Pulp sensitivity tests and radiographic analysis are the routine tests used to locate the involved teeth. Whereas the location of the sinus tract opening does not necessarily indicate the origin of the inflammatory exudate, tracking of the sinus tract with a gutta-percha point contributes to the final correct diagnosis [5,13]. However, a previous study reported an extraoral sinus tract that was misdiagnosed as an endodontic lesion. In the reported case, both periapical radiography and gutta-percha technique had failed to locate the origin of infection [14]. The newer 3-dimensional system, CBCT imaging, has been successfully applied in endodontics for improved detection of apical periodontitis and bone lesions [15], prediction of aberrant root canals [16] and evaluation of root canal preparation/obturation [15]. In our case report, we confirmed the size and extent of bone destruction around the apexes of tooth #47 via CBCT. Through 3D- reconstruction, the perforation of the lingual cortical plate was observed intuitively (Figure 1K).

Elimination of the infection is critical for the treatment of the draining sinus tract, either by endodontic therapy of restorable teeth or extraction of unrestorable teeth [5,17]. Excision of the sinus tract is not recommended, as most authors believe that the tract will heal once the primary cause is removed. A cutaneous sinus tract is a localized entity and antibiotics are usually not necessary for patients without systemic symptoms [5]. In the present case study, as tooth #47 was restorable and the patient was generally healthy without any systemic symptoms, nonsurgical root canal therapy was performed.

Thorough knowledge of both normal and abnormal root canal morphology contributes to successful endodontic treatment. The inability to locate, debride or fill all canals of the root canal system has been a major cause of post-treatment failure [18]. Mandibular second molars usually present with two roots (mesial and distal). However, clinicians must be aware that the mandibular second molar has a 0.38 - 2.8% chance, varying with different ethnic groups of a DL root (radix entomolaris) [19-21]. The majority of the DL roots in mandibular second molars have different degrees of curvature. The DL root is commonly located in the same buccolingual plane as the distobuccal root and can be “hidden” on the preoperative radiograph, which may result in the DL canal being left out during root canal treatment. The preoperative periapical radiograph from our patient did not reveal the presence of a DL root. However, using 3-dimensional CBCT imaging, we identified the presence of a DL root with significant curvature. The preparation of such a severely curved DL root canal can be an endodontic challenge and can lead to procedural errors [22]. Therefore, in this case report, the DL canal was explored by the pre-curved #6 K file before preparation and carefully prepared using the balance force technique with #6, 8, 10, 12, 15, 20 and 25 K-files, successively.

Sodium hypochlorite (NaOCl) is one of the most important and effective root canal irrigants due to its excellent bactericidal and tissue-dissolving properties. However, it also exhibits cytotoxicity and may cause severe tissue damage when extruded apically [23]. NaOCl accidents are far more likely to occur where there is significant periapical bone resorption involving perforation of the buccal or lingual cortical plate [24]. In our case report, the periapical bone loss around tooth #47 was so obvious that the perforation of lingual cortex could be seen on the CBCT images. The leakage of hydrogen peroxide from the sinus tract during irrigation also suggested that there was a high risk of NaOCl extrusion. Therefore, a great deal of caution was required for the usage of NaOCl. NaOCl exhibits less cytotoxicity at lower concentrations and reduced antibacterial effectiveness that can be compensated for by the use of large amounts of irrigant [25]. We therefore used 2.5% NaOCl followed by adequate irrigation of the canals instead of 5.25%. Closed-ended needles have been proven to cause significantly less apical pressure and irrigant extrusion when compared with open-ended needles [26]. Furthermore, short needle insertion depth and the absence of needle wedging also leads to decreased irrigant extrusion [27]. Accordingly, appropriate measures were taken to avoid NaOCl extrusion: (a) use an endodontic syringe with a side-vented opening without excessive force; (b) ensure that this needle is not be wedged into the root canal; (c) make certain that the needle is at least 2 – 3 mm short of its working length.

## Conclusion

Dental infection should be considered a primary cause of cutaneous facial sinus tracts. In cases with restorable teeth, elimination of the infection through endodontic treatment leads to resolution of the sinus tract. Thorough knowledge of aberrant root canal anatomy is critical for infection management during root canal therapy. NaOCl accidents should be avoided when periapical bone destruction is significant, and CBCT imaging enables better evaluation of periapical bone destruction when evaluating the safety of NaOCl use. CBCT imaging facilitates successful endodontic treatment by aiding the diagnosis of odontogenic cutaneous sinus tract and enabling better understanding of unusual canal morphology.

## Consent

Written informed consent was obtained from the patient for publication of this Case report and any accompanying images. A copy of the written consent is available for review by the Editor-in Chief of this journal.
